# Prenatal diagnosis of midgut volvulus by fetal MRI: a retrospective study

**DOI:** 10.3389/fped.2024.1442866

**Published:** 2024-12-23

**Authors:** Chenguang Kou, Yanfang Song, Duo Gao, Lixia Zhou

**Affiliations:** ^1^ Department of Medical Imaging, Second Hospital of Hebei Medical University, Shijiazhuang, China; ^2^ Department of Radiology, Hebei Provincial Hospital of Chinese Medicine, Shijiazhuang, China

**Keywords:** intestinal obstruction, midgut volvulus, meconium pseudocyst, prenatal diagnosis, magnetic resonance imaging

## Abstract

**Background:**

Fetal midgut volvulus is a rare disease, with a high risk of potentially life-threatening fetal complications.

**Purpose:**

The aim of this study was to retrospectively analyze the imaging findings of fetal midgut volvulus diagnosed by magnetic resonance imaging (MRI) and explore its value in non-invasive prenatal diagnosis.

**Methods:**

A retrospective collection of data from 156 fetuses suspected of intestinal obstruction by ultrasound examination in our hospital was conducted. All ultrasound examinations showed fetal intestinal dilation and fetal MRI diagnosis suspected midgut volvulus in 32 cases (32/156), of which 18 cases (18/32) that underwent surgical treatment in the neonatal period were confirmed to have midgut volvulus. MRI signs in the 18 fetuses with midgut volvulus were analyzed.

**Results:**

During MRI examination, all 18 fetuses showed gastric and/or intestinal dilatation, most of which showed different degrees of obstruction in T1-weighted images (WIs) and T2WIs, showing the “black and white sign” (14/18), “whirlpool sign” (10/18), and “coffee bean sign” (6/18). High signal intensity changes in diffusion-weighted imaging sequences were observed in intestinal tubes with ischemia and infarction. Direct signs of vascular torsion were observed in some cases (8/18). MRI signs indicated fetal midgut volvulus with hydramnios (16/18), meconium pseudocyst (7/18), meconium peritonitis (4/18), testicular hydrocele (3/18), and secondary pulmonary dysplasia (6/18). Operations confirmed the diagnosis of segmental midgut volvulus in 15 cases, complete midgut volvulus in 3 cases, and combined with intestinal atresia in 8 cases.

**Conclusion:**

Prenatal MRI plays an important role in the diagnosis of fetal midgut volvulus and the discovery of its complications, which can guide the treatment after birth and provide a reference for the prognosis.

## Introduction

Fetal intestinal obstruction is a relatively common congenital intestinal abnormality. Prenatal diagnosis is very important for prognostic evaluation and postnatal surgical treatment. Midgut volvulus is a rare cause of fetal intestinal obstruction. Congenital midgut volvulus represents a twisting of small bowel loops or the proximal colon around the mesenteric artery or its branches. It is a rare and life-threatening disease (1, 2). Left untreated, intestinal obstruction and hemorrhage from the necrotic bowel tissue will lead to intrauterine death. Congenital malrotation of the intestine leads to a long and narrow mesentery in the midgut, resulting in an unstable intestinal position. Therefore, it is relatively easy for the midgut to twist around the superior mesenteric artery, causing intestinal obstruction and tissue ischemic infarction. This is considered the main cause of midgut volvulus and intestinal ischemia and necrosis (3, 4).

Magnetic resonance imaging (MRI) is capable of multi-planar and multi-sequence imaging and excellent soft tissue contrast and avoids the risks of ionizing radiation. This makes it a useful tool for imaging the small bowel and diagnosing midgut volvulus in pregnancy (5) and is significantly superior to ultrasound (US) in the diagnosis of intestinal lesions. Intestinal wall, intestinal canal, and mesangial blood vessel changes can be observed by MRI because the intestine is full of fluid, thus, it can act as a natural contrast agent. MRI can provide more specific information for neonatal operations after birth and is helpful for clinical treatment and prognosis.

The imaging diagnosis of midgut volvulus is usually performed during the neonatal period. There are a few imaging studies on fetal midgut volvulus, most of which used ultrasound, and only a few used MRI. The aim of this work is to describe and specify the MRI signs for a prenatal diagnosis of fetal midgut volvulus based on MRI data from the largest retrospective case series published thus far.

## Materials and methods

### Population

This study received approval from our local research ethics committee under notification number 2016164. The review board waived patient informed consent for this study. This retrospective descriptive study reviewed all cases of fetal intestinal dilatation diagnosed by US between October 2014 and December 2020.

Inclusion criteria: (1) Suspected intestinal obstruction and midgut volvulus on fetal MRI. (2) Surgical treatment or exploration in our pediatric surgery department during the neonatal period and confirmed midgut volvulus. In cases of suspected intrauterine fetal death, postnatal fetal death, or artificial termination of pregnancy, midgut volvulus is confirmed by an autopsy.

Exclusion criteria: (1) Lack of fetal MRI scans. (2) Failure to undergo surgery or autopsy after birth and no clear diagnosis.

The collected data take into account the age of the pregnant women, the duration of the pregnancy, description of MRI scans, pregnancy outcome, and pediatric surgery data or results of a fetal autopsy. The data are reported as mean (m ± SD) or numbers and percentages [*n* (%)].

### Magnetic resonance imaging

Prenatal MRI was performed on a 1.5T MR system (Optima MR360; GE Medical Systems, America). Fetal axial, coronal, and sagittal MR images were obtained for each sequence. The fast imaging employing steady-state acquisition (FIESTA) sequence was as follows: repetition time (TR), 5.3 ms; rcho time (TE), 2.3 ms. T2-weighted images (T2WIs) included the single shot-fast spin echo (SSFSE) sequence: TR, 2,000 ms; TE, 140 ms. T1-weighted images (T1WIs) included the fast inversion recovery motion insensitive (FIRM) sequence: TR, 150 ms; TE, 4.2 ms. Diffusion-weighted imaging (DWI) used the following sequence: TR, 150 ms; TE, 75 ms; b = 0; 700 s/mm^2^. Section thickness and spacing were 5 mm, while the field of view (FOV) used was 380 mm × 380 mm.

## Results

A total of 156 fetuses were suspected of intestinal obstruction by ultrasound examination, of which 32 cases were diagnosed with midgut volvulus on MRI, and 18 cases (gestational age: 22–38 weeks) were confirmed to be midgut volvulus in neonatal operations after birth. Prenatal MRI imaging findings, clinical data, and final diagnosis by surgery were assessed in these 18 cases (Table 1). Of the 18 fetuses, 15 cases had gastric dilation (15/18) and 16 cases had intestinal dilatation (16/18) on MRI examination. In total, 14 cases had a “black and white sign” (14/18), 10 cases had a “whirlpool sign” (10/18), and 6 cases had a “coffee bean sign” (6/18). The ischemic infarction of the intestinal wall had high signal in the DWI sequences. Some cases showed direct signs of vascular torsion in T2WIs or DWI b = 0 sequences (8/18). MRI showed hydramnios fluid (16/18), meconium pseudocysts (7/18), meconium peritonitis (4/18), testicular hydroceles (3/18), and secondary pulmonary dysplasia (6/18) in the fetuses with midgut volvulus. The operations confirmed the diagnosis of segmental midgut volvulus in 15 cases, complete midgut volvulus in 3 cases, and combined with intestinal atresia in 8 cases.

**Table 1:** Summary of the patients’ clinical characteristics and MRI findings.

Patient ID	Age (years)	GA at scan (weeks)	Mode of delivery	MRI findings								Surgical findings or autopsy	Fetal outcome
				Whirlpool sign	Coffee bean sign	Black and white sign	Signals on DWI sequence	Gastric dilation	Bowel dilation	Hydramnios	Meconium pseudocyst		
1	28	35	V	+	−	+	H	+	+	+	−	Intraoperative: Ileum torsion; perforation with necrosis; meconium peritonitis, and severe intraperitoneal adhesion. Several segments of intestinal tubes were examined, with a total length of approximately 36 cm. Chronic mucosal inflammation, vasodilation, congestion, and bleeding were observed under microscope.	Died 10 days after surgery
2	24	33	CD	+	+	+	H	+	+	−	−	Intraoperative: Intestinal atresia with torsion; meconium peritonitis	Died 30 days after surgery
3	32	37	CD	+	+	+	H	+	+	+	+	Intraoperative: Ileal atresia and torsion; meconium pseudocyst, peritonitis	Died 21 days after surgery
4	25	34	V	+	−	+	H	+	+	+	−	Intraoperative: Neonatal small intestinal torsion; strangulated intestinal obstruction	Survival
5	33	36	CD	+	+	+	H	+	−	−	–	Intraoperative: Intestinal atresia with torsion; meconium peritonitis	Survival
6	26	32	CD	−	−	+	H	+	+	+	–	Intraoperative: Intestinal atresia with torsion, meconium peritonitis	Died 7 days after surgery
7	38	33	V	−	+	+	H	+	+	+	+	Intraoperative: Complete volvulus with intestinal necrosis; meconium pseudocyst	Died 2 days after surgery
8	21	35	CD	+	−	+	H	−	+	+	−	Intraoperative: Torsion of jejunum; meconium peritonitis	Died 150 days after surgery
9	24	29	V	−	−	+	L	+	+	+	+	Intraoperative: Complete intestinal atresia with volvulus; meconium pseudocyst	Died 3 days after surgery
10	31	25	V	−	−	+	L	+	−	+	−	Intraoperative: Segmental intestinal volvulus; dilatation of intestine	Survived
11	23	32	CD	+	+	−	L	+	+	+	+	Intraoperative: Complete intestinal volvulus; intestinal atresia; meconium pseudocyst	Died 7 days after surgery
12	30	26	V	+	−	−	H	−	+	+	+	Intraoperative: Congenital jejunal atresia; congenital malrotation of the intestine with torsion; congenital short small intestine	Survived
13	32	36	CD	−	+	+	H	−	+	+	+	Intraoperative: Segmental intestinal volvulus with ischemic necrosis; meconium peritonitis	Died 182 days after surgery
14	25	30	V	+	−	−	L	+	+	+	−	Intraoperative: Segmental intestinal volvulus	Survived
15	33	24	TOP	−	−	−	H	+	+	+	+	Intraoperative: Complete intestinal volvulus; intestinal dysplasia; meconium pseudocyst	Died
16	34	37	CD	−	−	+	H	+	+	+	−	Intraoperative: Segmental intestinal volvulus; meconium peritonitis	Survived
17	29	35	V	+	−	+	L	+	+	+	−	Intraoperative: Segmental intestinal volvulus	Survived
18	22	28	V	−	−	+	L	+	+	+	−	Intraoperative: Segmental intestinal volvulus	Survived
Total [m ± SD or *n* (%)]	28.33 ± 1.135	32.06 ± 0.9784	–	10/18 56%	6/18 33%	14/18 78%	12/18 67%	15/18 83%	16/18 89%	16/18 89%	7/18 39%	–	Mortality: 10/18 (56%)

GA, gestational age; V, vaginal delivery; CD, cesarean delivery; TOP, termination of pregnancy; H, high; L, low.

### Whirlpool sign

**Figure 1:**
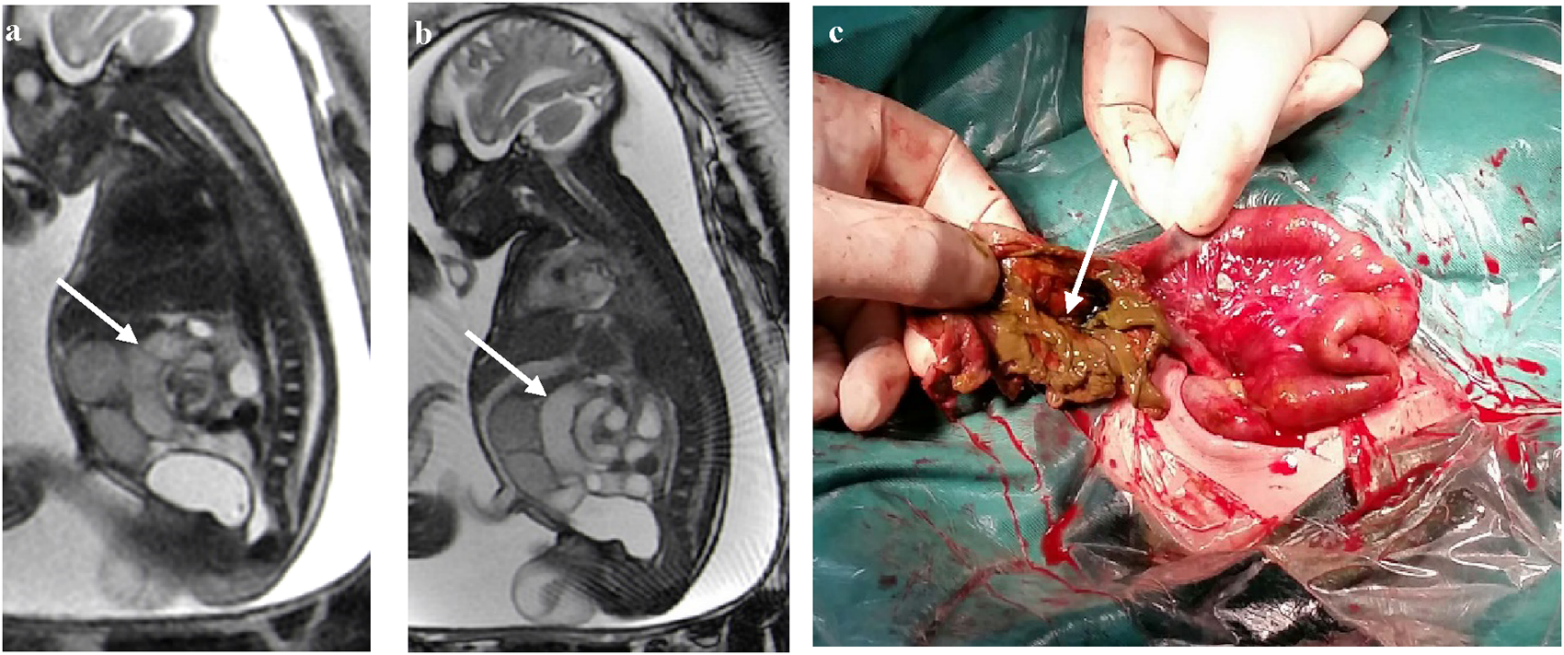
**(a–c)** In a case at 35 weeks of gestation, the bowel arrangement has changed to a whirlpool shape with vascular shadow in the center of the whirlpool indicated by a white arrow. Ileum torsion, perforation with necrosis, meconium peritonitis, and severe intraperitoneal adhesions were observed in the operation (white arrows).

A whirlpool sign, showing torsion of the intestine around the mesentery, is arranged in a swirl or concentric circle with the lumen dilated to different degrees. In the FIESTA and SSSFE sequences, most of the torsion and dilation of the lumen was less signalized than in the normal sequences. The surgical evaluations showed ileum torsion, perforation with necrosis, meconium peritonitis, and severe intraperitoneal adhesion (Figures 1a,b,c).

### Coffee bean sign

**Figure 2:**
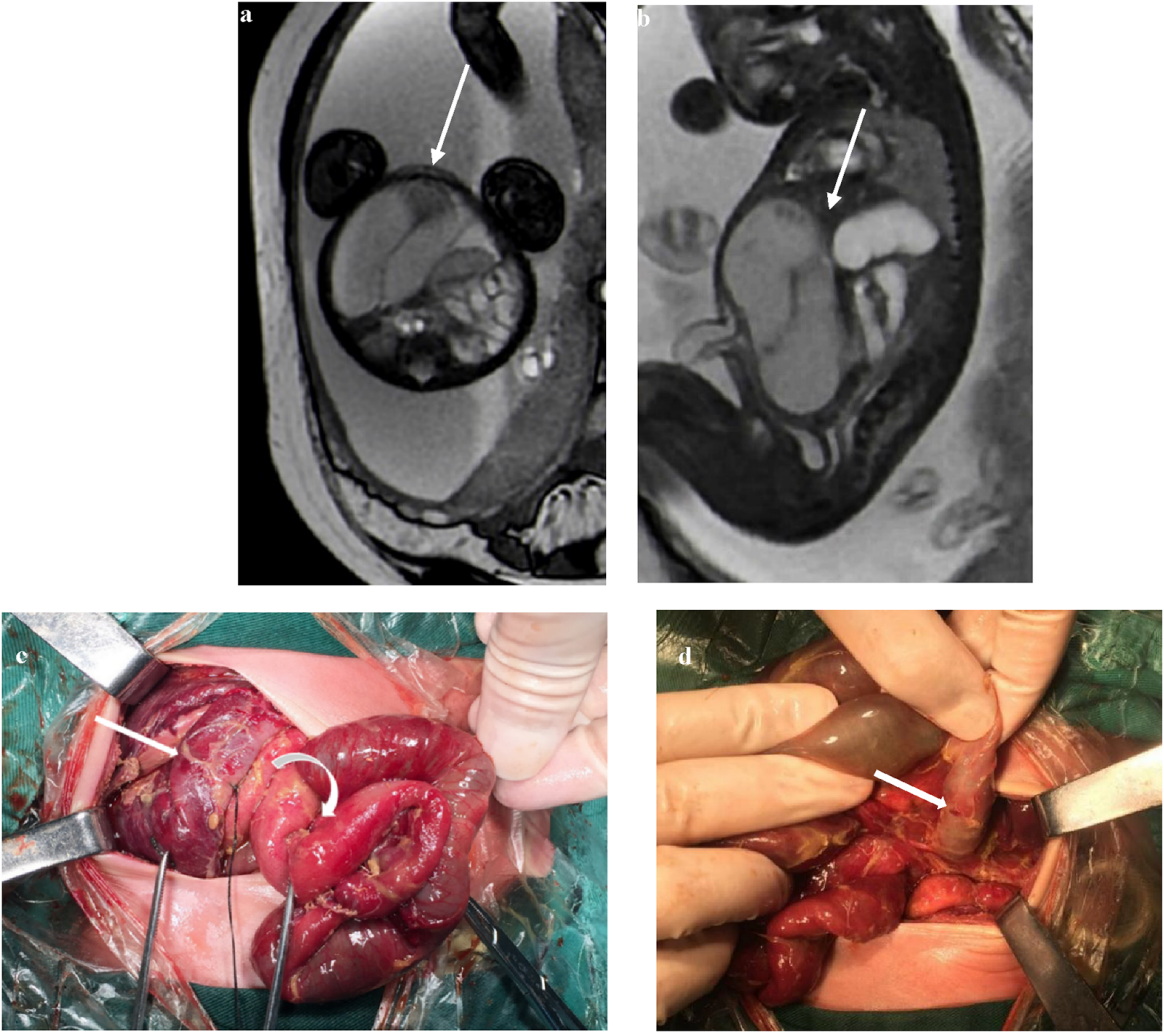
**(a)** At 37 weeks of gestation, a FIESTA sequence axis shows a dilated small intestine with a coffee bean sign (white arrow). **(b)** At 32 weeks of gestation, the sagittal image shows a dilated small intestine with a coffee bean sign. The intestinal wall is swollen and thickened (white arrow). **(c)** The small intestine is ischemic after torsion (straight arrow) with a relatively normal small intestine (curved arrow). **(d)** Perforation (straight arrow) can be seen in the ischemic intestinal wall.

The closed loop of the intestine presents an elliptical shape with obvious dilation and accumulation of fluid. The adjacent intestinal walls become swollen and thickened, and they converge to form a dense linear shadow, resembling coffee beans as a whole (Figures 2a,b). A twisted and dilated ischemic jejunum with a relatively normal intestinal canal adjacent to the small intestine could be seen during surgery in a newborn with midgut volvulus. Perforation was seen in the intestinal wall with torsion of the necrotic jejunum (Figures 2c,d).

### Black and white sign

**Figure 3:**
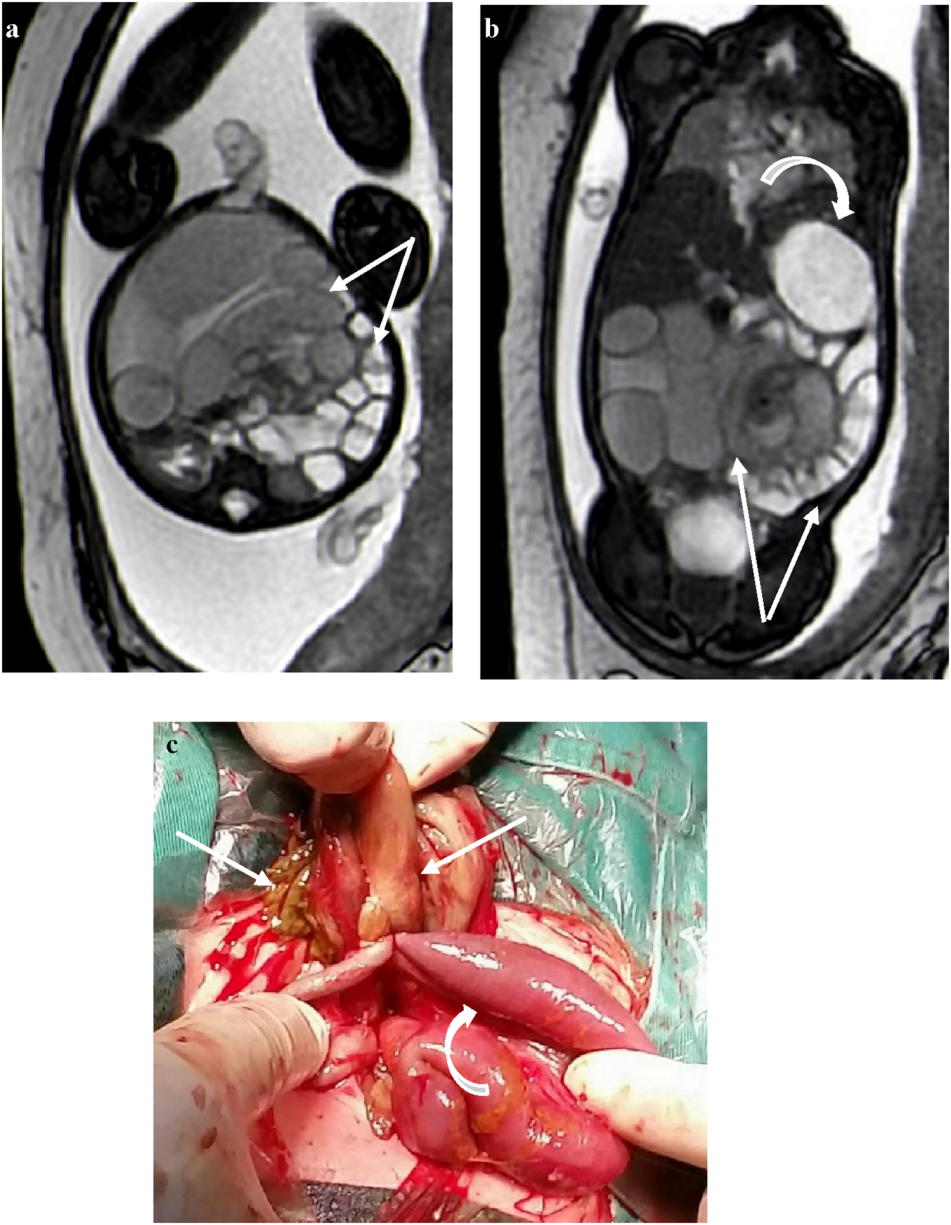
At 35 weeks of gestation, **(a)** the transverse section of a FIESTA sequence: the signal intensity of the dilated intestine torsion is reduced, and the relatively normal intestines around it show high signal intensity with sharp contrast, showing a “black and white sign” (white arrows); **(b)** the gastric vesicle is enlarged (crooked arrow). **(c)** Ischemic necrotic intestine (straight arrow) and adhesive normal intestine (crooked arrow).

In most cases, the contrast between the obstructed intestinal tubes and the relatively normal intestinal tubes on T1WI and T2WI sequences was sharp with black and white signs (Figures 3a,b). The abdominal intestinal tubes of normal fetuses were filled with amniotic fluid, showing hypersignals in the T2WIs and hyposignals in the T1WI. The T2WI signal intensity was low in the torsion and obstruction areas of the lesion, and the T1WI signal intensity was high in some of the infarcted intestines, which was considered to be caused by meconium deposition combined with ischemia and hemorrhage.

### Signals on the DWI sequence

**Figure 4:**
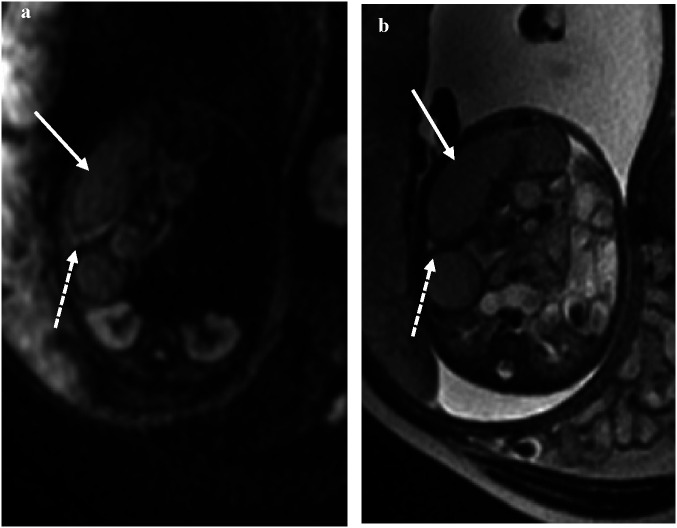
At 35 weeks of gestation, **(a)** the DWI signal intensity of the obstruction of the intestine is higher than that of the lumen, suggesting ischemia. **(b)** The corresponding intestinal lumen showed slightly lower signal intensity on T2WI.

The DWI sequences (b = 700) showed hyperintensity for torsion with infarction and intestinal wall swelling and thickening (Figures 4a,b). The high signal intensity of DWI in the volvulus obstruction suggests hemorrhage, necrosis, or increased protein content in the lumen, with higher signal intensity in the intestinal wall indicating ischemic necrosis.

**Figure 5:**
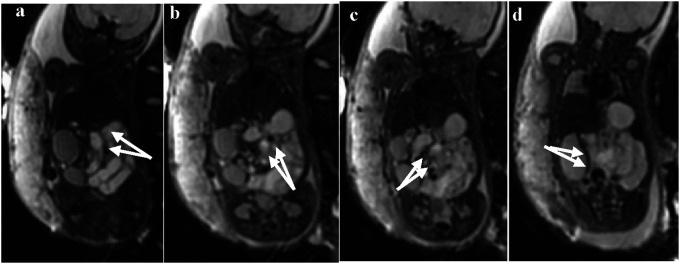
**(a–d)** Visible signs of mesenteric vessel malrotation and even volvulus with vessel lumen dilatation indicated by white arrows.

In some cases, the b = 0 sequence of DWI showed signs of mesenteric vessel malrotation (Figures 5a–d) in which the mesenteric vessels were spirally rotated with the volvulus.

## Discussion

Fetal midgut volvulus is rare, and only a few cases of prenatal diagnosis have been reported (6–9). Most cases of segmental volvulus occur with obstruction which is usually associated with intestinal rotation anomalies (10, 11). However, the pathogenesis of volvulus without malrotation remains unknown, and might be related to congenital developmental defects such as intestinal atresia, gastroschisis, and meconium plug syndrome (12). Black et al. (13) demonstrated that segmental mesenteric defects are common in this group, with an etiological role. In a study of patients with volvulus without malrotation, we hypothesize that volvulus might be caused by meconium plug syndrome without malrotation, because a dilated bowel contains large amounts of meconium, while the distal ileum and colon are distended with fluid. In addition, the relationship between the superior mesenteric vein (SMV)-superior mesenteric artery (SMA) and the anatomy of the ligament of Treitz are maintained.

Prenatal screening for gastrointestinal (GI) abnormalities is based on ultrasound examination in pregnancies with a duration of more than 20 weeks. However, in current practice, sonographic findings lack the specificity to address questions pertaining to the precise number of obstructions and their locations and the length of the intestinal tract involved in the obstruction (14). Fetal ultrasound examination has many limitations and is easily affected by fetal position and maternal intestinal gas. It has poor penetration and low spatial resolution. Since the fetal small intestine is filled with amniotic fluid, fetal intestinal MRI has a good natural contrast, similar to that of a gastrointestinal examination. Multiple systems can be observed in one scan, and its localization and diagnostic value are significantly better than fetal ultrasound. Therefore, fetal MRI can be used as a more accurate diagnostic examination when a prenatal ultrasound suggests a lesion of the gastrointestinal tract. A study demonstrated a good correlation between MRI and surgical findings in distal small bowel obstruction (15). MRI patterns of normal and abnormal GI tracts have also been recently described (16–18). Furthermore, MRI seems to be more sensitive than US in detecting the presence of meconium (19).

According to previous reports, fetal segmental volvulus may show specific signs such as the “coffee bean sign,” “banana sign,” and “whirlpool sign” (dilated bowel loops forming a typical convoluted mass) (20, 21), consistent with our findings. The whirlpool sign is a very sensitive (89%) and specific (92%) postnatal sign (22), even if not fully pathognomonic of intestinal volvulus. Shimanuki et al. (23) proposed that the clockwise whirlpool sign is almost 100% specific for intestinal volvulus, while the anticlockwise whirlpool sign is non-specific and is found in cases without volvulus. Moreover, the “coffee bean sign” is specific to a certain stage of ileus, strongly suggesting the diagnosis of volvulus. Non-specific findings include cystic abdominal mass, dilated bowel loops, peritoneal calcifications, ascites, and polyhydramnios, which could reflect prenatal segmental volvulus.

Fetal MRI intestinal signals vary in the gestational period and are related to the content of amniotic fluid and meconium in the intestine (19). Meconium contains high levels of protein and paramagnetic mineral components (iron, copper, and manganese) that can explain the high T1 and low T2 signal intensities observed (19). Meconium is produced after 13 weeks, and slowly migrates from the small bowel to the colon and rectum in normal fetuses during the first trimester. After 20 weeks of gestation, the colon and rectum constitute a meconium reservoir, while the small bowel is filled with ingested amniotic fluid. This explains the normal fetal GI tract signals, with the colon and rectum exhibiting meconium-like high-intensity signals on T1WIs and being poorly visualized on T2WIs. In comparison, the stomach and small bowel have high-intensity signals on T2WI because of their fluid content. The abnormal shape of continuous blood vessels was observed in the DWI sequences (b = 0), such as helical changing, abnormal dilation, and signal abnormality. This is similar to the morphological manifestations such as vascular vortexes observed in ultrasound, but, currently, fetal MRI cannot reflect the direction and velocity of blood flow in the mesenteric vessels. DWI sequencing has a fast imaging speed and is also a commonly used fetal scanning sequence. The small intestine and colon of a normal fetus exhibit low signal intensity in the DWI sequence. When volvulus occurs, the intestinal blood vessels are occluded, resulting in ischemic necrosis of the intestine. DWI sequences can reflect the degree of ischemic necrosis of the intestinal wall, and the signals from the intestinal wall and intestinal contents will change accordingly in different segments. In DWI sequences, the ischemic intestinal wall has a high signal intensity, which is related to cytotoxic edema caused by ischemic necrosis. When intestinal necrosis occurs, the blood in the intestinal cavity mixes with inflammatory substances and protein accumulation, leading to limited diffusion of water molecules and causing an increase in DWI signal intensity in the intestinal lumen. Simultaneously, secondary inflammation of the intestinal wall can also exacerbate the increased DWI signal intensity in the intestinal wall. Between 20 and 24 gestational weeks, the main contents in the terminal small intestine and colorectum are meconium. The minerals in meconium, such as copper, iron, and manganese, have a paramagnetic effect, which can shorten the T1 time and show high T1 signal intensity. The content of the esophagus, stomach, and most of the small intestine is amniotic fluid, showing long T1 and T2 signals. After that, there is a low signal intensity in the small intestine from the amniotic fluid. Therefore, a high-intensity T1 signal appears in the small intestine of the fetus in the middle and late trimester, and we should be alert to lesions such as obstruction and meconium pseudocysts. Thus, a fetal intraperitoneal cystic mass that shows high T1 and low T2 signal intensities is considered pathognomonic of a walled-off collection secondary to *in-utero* bowel perforation (meconium pseudocyst) (16, 19).

The diagnosis of midgut volvulus does not necessarily require an immediate intervention. If the fetal intrauterine status and dilated bowel are stable, close fetal monitoring and regular scans (each 1–3 weeks) can be planned. However, in the case of intestinal hematocele, perforation, and peritonitis, or the absence of intestinal peristalsis, the pregnancy should be interrupted without delay (7). The post-operative prognosis of these patients remains variable and depends on the site and extent of the bowel involvement and the patient’s gestational age (24).

Prenatal MRI has excellent diagnostic value for the diagnosis of volvulus as many types of direct imaging signs can be observed in a variety of complications, such as abdominal lesions (intestinal dilation, meconium pseudocysts, meconium peritonitis) and chest lesions (e.g., secondary pulmonary hypoplasia). It has advantages over ultrasound as it can roughly indicate the site of obstruction, find meconium pseudocysts, and indicate the degree and extent of ischemic necrosis of the intestinal wall.

## Conclusions

We found that the MRI diagnosis of fetal midgut volvulus is consistent with intraoperative findings, and multiple imaging signs can indicate midgut volvulus. MRI is a useful supplement in the diagnosis of midgut volvulus, allowing for detailed imaging of intestinal lesions with high resolution, and the dilatation, stricture, assessment of meconium distribution, and abnormal arrangement of the intestine can be directly observed. High signal intensity in DWI can indicate ischemia of the intestinal wall. It has an important role in guiding obstetric management and neonatal treatment.

## Data Availability

The original contributions presented in the study are included in the article/Supplementary Material, further inquiries can be directed to the corresponding author.
