# Correction: Prenatal diagnosis of midgut volvulus by fetal MRI: a retrospective study

**DOI:** 10.3389/fped.2026.1857727

**Published:** 2026-04-30

**Authors:** Chenguang Kou, Yanfang Song, Duo Gao, Lixia Zhou

**Affiliations:** 1 Department of Medical Imaging, Second Hospital of Hebei Medical University, Shijiazhuang, China; 2 Department of Radiology, Hebei Provincial Hospital of Chinese Medicine, Shijiazhuang, China

**Keywords:** intestinal obstruction, midgut volvulus, meconium pseudocyst, prenatal diagnosis, magnetic resonance imaging

A Correction on Prenatal diagnosis of midgut volvulus by fetal MRI: a retrospective study By Kou C, Song Y, Gao D and Zhou L (2024). Front. Pediatr. 12:1442866. doi: 10.3389/fped.2024.1442866

Incorrect funding

The funder Medical Science Research Project of Hebei, [20220961] to Yanfang Song was erroneously omitted.

The original version of this article has been updated.

